# Diagnostic Accuracy of the Cepheid 3-gene Host Response Fingerstick Blood Test in a Prospective, Multi-site Study: Interim Results

**DOI:** 10.1093/cid/ciab839

**Published:** 2021-09-22

**Authors:** Jayne S Sutherland, Gian van der Spuy, Awa Gindeh, Nguyen Thuy Thuong Thuong, AnnRitah Namuganga, Olumuyiwa Owolabi, Harriet Mayanja-Kizza, Mary Nsereko, Guy Thwaites, Jill Winter, Hazel M Dockrell, Thomas J Scriba, Annemieke Geluk, Paul Corstjens, Kim Stanley, Tracy Richardson, Jane A Shaw, Bronwyn Smith, Stephanus T Malherbe, Gerhard Walzl, TrENDx-TB Consortium, Jayne Sutherland, Olumuyiwa Owolabi, Amie Secka, Georgetta Daffeh, Awa Gindeh, Joseph Mendy, Binta Sarr, Abi-Janet Riley, Alhaji Jobe, Monica Davies, Kairaba Kanyi, Momodou Jallow, Salieu Barry, Ousainou Cham, Esin Nkereuwem, Gerhard Walzl, Stephanus Malherbe, Bronwyn Smith, Gian van der Spuy, Kim Stanley, Jane Shaw, Alicia Chetram, Tracy Richardson, Marika Finn, Andriette Hiemstra, Novel Chegou, Helena Kuivaniemi, Gerard Tromp, Susanne Tonsing, Elizma Smit, Balie Carstens, Harriet Mayanja-Kizza, Mary Nsereko, AnnRitah Namuganga, Sophie Nalukwago, Joseph Akol, Dorcas Lamunu, Michael Ordie, Guy Thwaites, Thuong Nguyen, Van Le, Son Vo Thanh, Hau Nguyen Thi, Ha Vu Thi Ngoc, Ngoc Le Hong, John Belisle, Karen Dobos, Hazel Dockrell, Thomas Scriba, Mark Hatherill, Kate Hadley, Justin Shenje, Stanley Kimbung, Humphrey Mulenga, Rachel Oelofse, Nicole Bilek, Elma van Rooyen, Simba Mabwe, Paul Corstjens, Annemieke Geluk, Elisa Tjon Kon Fat, Louise Pierneef, Anouk van Hooij, Jill Winter, Morten Ruhwald, Emmanuel Moreau, Adam Penn-Nicholson, Claudia Schacht, Julia Büech, Malte Streitz

**Affiliations:** Vaccines and Immunity Theme, Medical Research Council (MRC) Unit, The Gambia at the London School of Hygiene and Tropical Medicine, Banjul, The Gambia; Department of Science and Technology National Research Foundation (DST-NRF) Centre of Excellence for Biomedical Tuberculosis Research, South African Medical Research Council Centre for Tuberculosis Research, Division of Molecular Biology and Human Genetics, Faculty of Medicine and Health Sciences, Stellenbosch University, Cape Town, South Africa; Vaccines and Immunity Theme, Medical Research Council (MRC) Unit, The Gambia at the London School of Hygiene and Tropical Medicine, Banjul, The Gambia; Oxford University Clinical Research Unit, Ho Chi Minh City, Vietnam; Centre for Tropical Medicine and Global Health, Nuffield Department of Medicine, University of Oxford, Oxford, UK; Makerere University, Kampala, Uganda; Vaccines and Immunity Theme, Medical Research Council (MRC) Unit, The Gambia at the London School of Hygiene and Tropical Medicine, Banjul, The Gambia; Makerere University, Kampala, Uganda; Makerere University, Kampala, Uganda; Oxford University Clinical Research Unit, Ho Chi Minh City, Vietnam; Centre for Tropical Medicine and Global Health, Nuffield Department of Medicine, University of Oxford, Oxford, UK; Catalysis Foundation, Berkeley, California, USA; London School of Hygiene and Tropical Medicine, London, United Kingdom; South African Tuberculosis Vaccine Initiative, Institute of Infectious Disease and Molecular Medicine, Division of Immunology, Department of Pathology, University of Cape Town, South Africa; Department of Infectious Diseases, Leiden University Medical Center, The Netherlands; Department of Cell and Chemical Biology, Leiden University Medical Center, The Netherlands; Department of Science and Technology National Research Foundation (DST-NRF) Centre of Excellence for Biomedical Tuberculosis Research, South African Medical Research Council Centre for Tuberculosis Research, Division of Molecular Biology and Human Genetics, Faculty of Medicine and Health Sciences, Stellenbosch University, Cape Town, South Africa; Department of Science and Technology National Research Foundation (DST-NRF) Centre of Excellence for Biomedical Tuberculosis Research, South African Medical Research Council Centre for Tuberculosis Research, Division of Molecular Biology and Human Genetics, Faculty of Medicine and Health Sciences, Stellenbosch University, Cape Town, South Africa; Department of Science and Technology National Research Foundation (DST-NRF) Centre of Excellence for Biomedical Tuberculosis Research, South African Medical Research Council Centre for Tuberculosis Research, Division of Molecular Biology and Human Genetics, Faculty of Medicine and Health Sciences, Stellenbosch University, Cape Town, South Africa; Department of Science and Technology National Research Foundation (DST-NRF) Centre of Excellence for Biomedical Tuberculosis Research, South African Medical Research Council Centre for Tuberculosis Research, Division of Molecular Biology and Human Genetics, Faculty of Medicine and Health Sciences, Stellenbosch University, Cape Town, South Africa; Department of Science and Technology National Research Foundation (DST-NRF) Centre of Excellence for Biomedical Tuberculosis Research, South African Medical Research Council Centre for Tuberculosis Research, Division of Molecular Biology and Human Genetics, Faculty of Medicine and Health Sciences, Stellenbosch University, Cape Town, South Africa; Department of Science and Technology National Research Foundation (DST-NRF) Centre of Excellence for Biomedical Tuberculosis Research, South African Medical Research Council Centre for Tuberculosis Research, Division of Molecular Biology and Human Genetics, Faculty of Medicine and Health Sciences, Stellenbosch University, Cape Town, South Africa

**Keywords:** tuberculosis, diagnostics, Cepheid 3-gene host-response prototype, fingerstick blood, triage test

## Abstract

**Background:**

The development of a fast and accurate, non-sputum-based point-of-care triage test for tuberculosis (TB) would have a major impact on combating the TB burden worldwide. A new fingerstick blood test has been developed by Cepheid (the Xpert MTB Host Response [MTB-HR] prototype), which generates a “TB score” based on messenger RNA (mRNA) expression of 3 genes. Here we describe the first prospective findings of the MTB-HR prototype.

**Methods:**

Fingerstick blood from adults presenting with symptoms compatible with TB in South Africa, The Gambia, Uganda, and Vietnam was analyzed using the Cepheid GeneXpert MTB-HR prototype. Accuracy of the Xpert MTB-HR cartridge was determined in relation to GeneXpert Ultra results and a composite microbiological score (GeneXpert Ultra and liquid culture) with patients classified as having TB or other respiratory diseases (ORD).

**Results:**

When data from all sites (n = 75 TB, 120 ORD) were analyzed, the TB score discriminated between TB and ORD with an area under the curve (AUC) of 0.94 (95% confidence interval [CI], .91–.97), sensitivity of 87% (95% CI, 77–93%) and specificity of 94% (88–97%). When sensitivity was set at 90% for a triage test, specificity was 86% (95% CI, 75–97%). These results were not influenced by human immunodeficiency virus (HIV) status or geographical location. When evaluated against a composite microbiological score (n = 80 TB, 111 ORD), the TB score was able to discriminate between TB and ORD with an AUC of 0.88 (95% CI, .83–.94), 80% sensitivity (95% CI, 76–85%) and 94% specificity (95% CI, 91–96%).

**Conclusions:**

Our interim data indicate the Cepheid MTB-HR cartridge reaches the minimal target product profile for a point of care triage test for TB using fingerstick blood, regardless of geographic area or HIV infection status.

## Teaser

This study is the first prospective evaluation of the Cepheid MTB-HR test using fingerstick blood in a multi-site cohort. Interim results indicate the test reaches the WHO TPP for a TB Triage test regardless of geographical location and HIV status.

Development of a noninvasive, non-sputum-based point-of care test for tuberculosis (TB) is essential for providing timely diagnosis and reducing morbidity and mortality. While there are an estimated 10 million new cases of TB each year, close to 4 million of these are missed due to lack of adequate diagnostics, further fueling *Mycobacterium tuberculosis* (Mtb) transmission [1]. In low- and middle-income countries (LMIC), inadequate diagnostic tools not only lead to underdiagnosis but also misdiagnosis, aberrant use of antibiotics and a large delay between symptom onset and start of treatment. During the coronavirus disease 2019 (COVID-19) pandemic, this delay in diagnosis has been further exacerbated due to redirection of healthcare needs away from TB, lockdown limiting access to facilities, reduced TB testing in favor of COVID-19 testing, and increased stigma significantly affecting healthcare-seeking behaviors.

Current gold-standard tests for TB based on detection of the pathogen, are limited by cost, infrastructure requirements, and lack of sensitivity particularly in people living with human immunodeficiency virus (PLHIV) and children who tend to have paucibacillary disease. Several point-of-care (POC) tests are in development, including detection of blood host transcriptomic or protein markers or bacterial antigens secreted in the urine. Recent studies have identified a urinary lipoarabinomannan (LAM) test with up to 75% sensitivity and excellent specificity in individuals living with HIV and those who are not [2, 3].

Host transcriptomic signatures also hold great promise for development of diagnostic and/or triage tests. In 2016, Sweeney et al identified a combinatory score (TB score) based on blood messenger RNA (mRNA) expression levels of 3 differentially expressed genes (guanylate binding protein 5 [GBP5], dual specificity phosphatase 3 [DUSP3], and Krüppel-like factor 2 [KLF2]), for discrimination between active TB and other diseases [4] and was found to approach the TPP for a non-sputum-based triage test across 3 independent prospective cohorts [5]. Both GBP5 and DUSP3 are involved in the pro-inflammatory response and are upregulated in TB: inducible GBP5 has been shown to mediate the antiviral interferon response during influenza A virus infection [6] and human immunodeficiency virus type 1 (HIV-1) [7], although DUSP3 plays a nonredundant role as a regulator of innate immune responses by mechanisms involving the control of ERK1/2 activation, tumor necrosis factor (TNF) secretion, and macrophage polarization [8]. In contrast, KLF2, which regulates the inflammatory response by inhibiting the activation of monocytes [9], is downregulated in TB. This 3-gene signature has been incorporated into an automated qPCR test using the GeneXpert platform (Cepheid, USA). This Xpert *Mycobacterium tuberculosis* Host Response (MTB-HR) prototype quantifies the expression of the 3 transcripts in a whole-blood sample and then computes a TB score based on cycle threshold (Ct) values using an in-built algorithm. A recent publication analyzed the performance of the Xpert-MTB-HR-Prototype cartridge on stored PAXgene samples from individuals living with HIV from South Africa and Peru and showed good classification as a triage test [10], but performance in another retrospective study in Brazil failed to reach the target product profile (TPP) criteria for a triage test [11].

The aim of this study was to perform prospective evaluation of the Xpert-MTB-HR-Prototype against GeneXpert Ultra and a composite microbiological score (Xpert Ultra and culture). Our interim results indicate the MTB-HR-prototype is the first POC test to reach WHO TPP for a triage test for TB.

## METHODS

### Ethics Statement

Local ethics approval was obtained through the Medical Research Council (MRC)/London School of Hygiene and Tropical Medicine (LSHTM)/Gambian government joint ethics committee (MRCG at LSHTM); the Oxford Tropical Research Ethics Committee, the institutional review board at Pham Ngoc Thach Hospital, and the ethics committee of the Ministry of Health, Vietnam (OUCRU); the Stellenbosch University Health Research Ethics Committee (SUN) and the Uganda National Council for Science and Technology (MAK). Written informed consent was obtained from all participants prior to sample collection. All participants had blood sampling performed prior to knowledge of TB status. TB status was determined using GeneXpert Ultra (Cepheid, USA) and Liquid culture (MGIT; Becton Dickinson, USA).

### Patient Recruitment and Classification

Patients were consecutively recruited from local health clinics at each of the sites. Inclusion criteria included cough > 2 weeks and at least one other symptom suggestive of TB (ie, weight loss, hemoptysis, night sweats, fever). Sputum was taken for microbiological analysis and fingerstick blood for host gene response analysis. Patients with either a positive GeneXpert Ultra result or a positive culture result were classified as having TB, whereas those with both negative GeneXpert Ultra and culture were considered to have other respiratory diseases (ORD). However, due to the possible detection of false-positive results with GeneXpert Ultra [12], patients with a reading of “Trace” on GeneXpert Ultra who were culture positive were considered to have TB. Patients negative for both Xpert Ultra and culture were considered to have ORD. No patients with a positive GeneXpert Ultra but negative culture had prior TB.

### GeneXpert MTB Host-Response Analysis

In total, 200 µL of fingerstick blood (FSB) was collected with a minivette containing an anticoagulant (EDTA, Becton Dickinson, USA), transferred into an EDTA-microtainer (Becton Dickinson, USA), and inverted to mix. And 100 µL of the sample was added to the Cepheid MTB Host Response (MTB-HR) cartridge (donated by Cepheid, USA) and loaded into the GeneXpert machine. Ct values for individual genes (DUSP, GBP5 and KLF2) were obtained together with a TB score determined by: (Ct GBP5 + Ct DUSP3)/2 − Ct KLF2.^4^ If an invalid result was obtained, a second aliquot of 100 µL was added to a new cartridge and the sample retested.

### HIV Testing

All participants without known HIV infection received voluntary counseling and testing using a rapid fingerstick blood HIV-1/2 test (Alere, USA). If the rapid test was positive, the result was confirmed either with serology testing or a second rapid test. CD4 counts were determined for all individuals living with HIV at baseline.

### Statistical Analysis

Data were analyzed using GraphPad Prism v8.1 (Software MacKiev, USA) or R (version 4.1.0) with the pROC package (version 1.17.0.1). Mann-Whitney *U* tests were performed to compare Ct values for the transcripts of interest and the TB scores between total TB and ORD patients and at individual sites. Receiver operator characteristic (ROC) curves were used to determine the accuracy of each gene for the classification of TB, including area under the ROC curve (AUC), sensitivity, and specificity. A χ ^2^ test was used to compare sex, prior TB, and HIV status between the groups. WHO thresholds for a triage test are minimum 90% sensitivity, 70% specificity; ideal 95% sensitivity, 80% specificity [13]. Youdenʼs index was used to determine optimal sensitivity and specificity for the full cohort. Values for sensitivity and specificity in terms of WHO TPP were estimated using 200 repeats of 5-fold cross-validation. For each hold-out fold, specificity was determined from the training foldsʼ ROC curve at the lowest sensitivity ≥ 0.9 for a triage test. Mean sensitivity and the 95% confidence interval (CI) were calculated from the 1000 sensitivities thus obtained. Confidence intervals for ROC curve AUCs were obtained via 5000 bootstrap replicates.

## RESULTS

### Participant Information

We recruited 224 participants (from a total of 1200 expected participants for all sites) between December 2020 and May 2021. Of these, 28 participants did not have results for the Cepheid MTB-HR cartridges as recruitment began prior to obtaining the cartridges, and 1 participant did not have a GeneXpert Ultra result. Data from the remaining 195 participants are presented.

We analyzed the MTB-HR-protototype using fingerstick blood samples from a total of 75 patients with Xpert ultra positive results (TB) and 120 with Xpert ultra negative results (ORD) (Table 1). 20% of the TB group were females compared to 43% of the ORD group (*P* = .019; Table 1). Additionally, 5.3% of the TB group were living with HIV compared to 17% of the ORD group (not significant [NS]) with a median (interquartile range [IQR]) of 379 [90–793] CD4 cells/µL for TB and 344 [179–689] CD4+ T cells/µL for ORD (NS). No significant difference in age was seen between the groups with median (IQR) of 30 (26–38) years for the TB group and 36 (28–45) years for the ORD group (Table 1). Two patients (3%) in the TB group and 8 (7%) in the ORD group had a history of prior TB (NS; Table 1). For the GeneXpert Ultra positive (TB) group, 51% had a reading of “high,” 20% of “medium,” 19% of “low,” and 10% of “trace or very low” (Table 1).

**Table 1.** Patient Information

	Mtb Detected	Mtb Not Detected	*P* Value
n	75	120	
Age, median (IQR)	30 (26–38)	36 (28–45)	NS
Females, n (%)	20 (27)	52 (43)	.019
HIV positive, n (%)	4 (5.3)	17 (14)	NS
CD4, median (IQR)	379 (90–793)	344 (179–689)	NS
Prior TB, n (%)	2 (3)	8 (7)	NS
Xpert ultra reading			
Trace, n (%)	7 (9)	NA	
Very low, n (%)	1 (1)	NA	
Low, n (%)	14 (19)	NA	
Medium, n (%)	15 (20)	NA	
High, n (%)	38 (51)	NA	
Liquid culture			
Positive	68	8	
Negative	2	110	
Contaminated	3	1	
NA	2	1	

Abbreviations: CD4, count, cells/µL blood (only performed in PLHIV); HIV, human immunodeficiency virus; IQR, interquartile range; Mtb, *Mycobacterium tuberculosis*; NA, not applicable; NS, not significant; ORD, other respiratory diseases; TB, tuberculosis.

### Analysis of Data From All Sites

**Figure 1.**
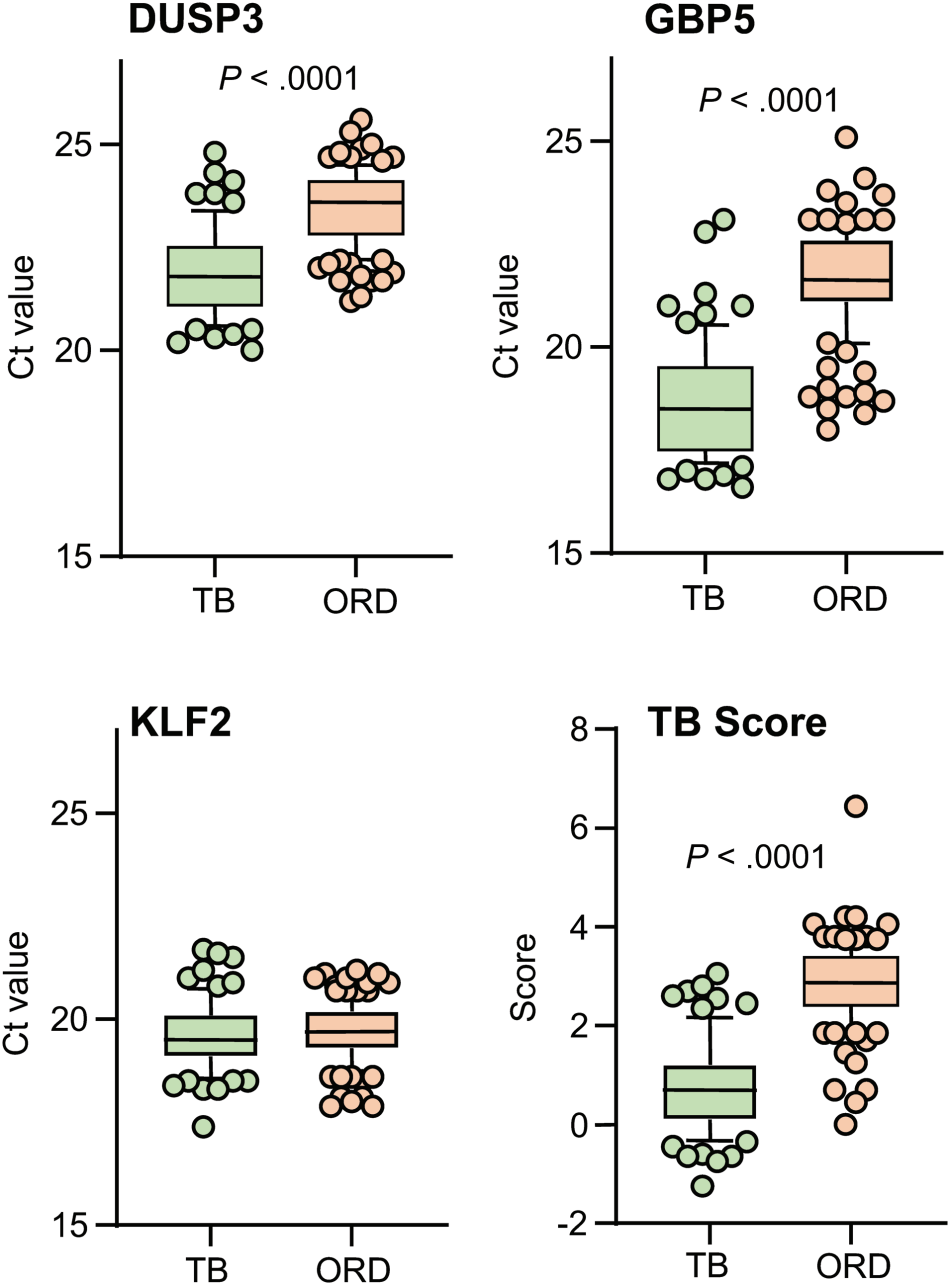
Xpert MTB-HR prototype test analysis in TB and ORD patients detected by GeneXpert Ultra. (*A*) DUSP3, (*B*) GBP5, (*C*) KLF2, (*D*) TB score. Data were analyzed using Mann-Whitney *U* test. Bars indicate 10–90% percentile, line indicates median, and dots indicate outliers. Ct value derived from the GeneXpert machine. Abbreviations: Ct, cycle threshold; DUSP3, dual specificity phosphatase 3; GBP5, guanylate binding protein 5; KLF2, Krüppel-like factor 2; MTB-HR, MTB-Host Response; ORD, other respiratory disease; TB, tuberculosis.

Analysis of individual genes for all participants, showed significantly lower Ct values (higher input mRNA) for both DUSP3 and GBP5 genes in TB compared to ORD patients (*P* < .0001 for both; Figures 1A, 1B). No difference in KLF2 was seen between the groups (Figure 1C) thus when the TB score was automatically calculated, it was significantly lower in TB compared to ORD patients (*P* < .0001; Figure 1D).

**Figure 2.**
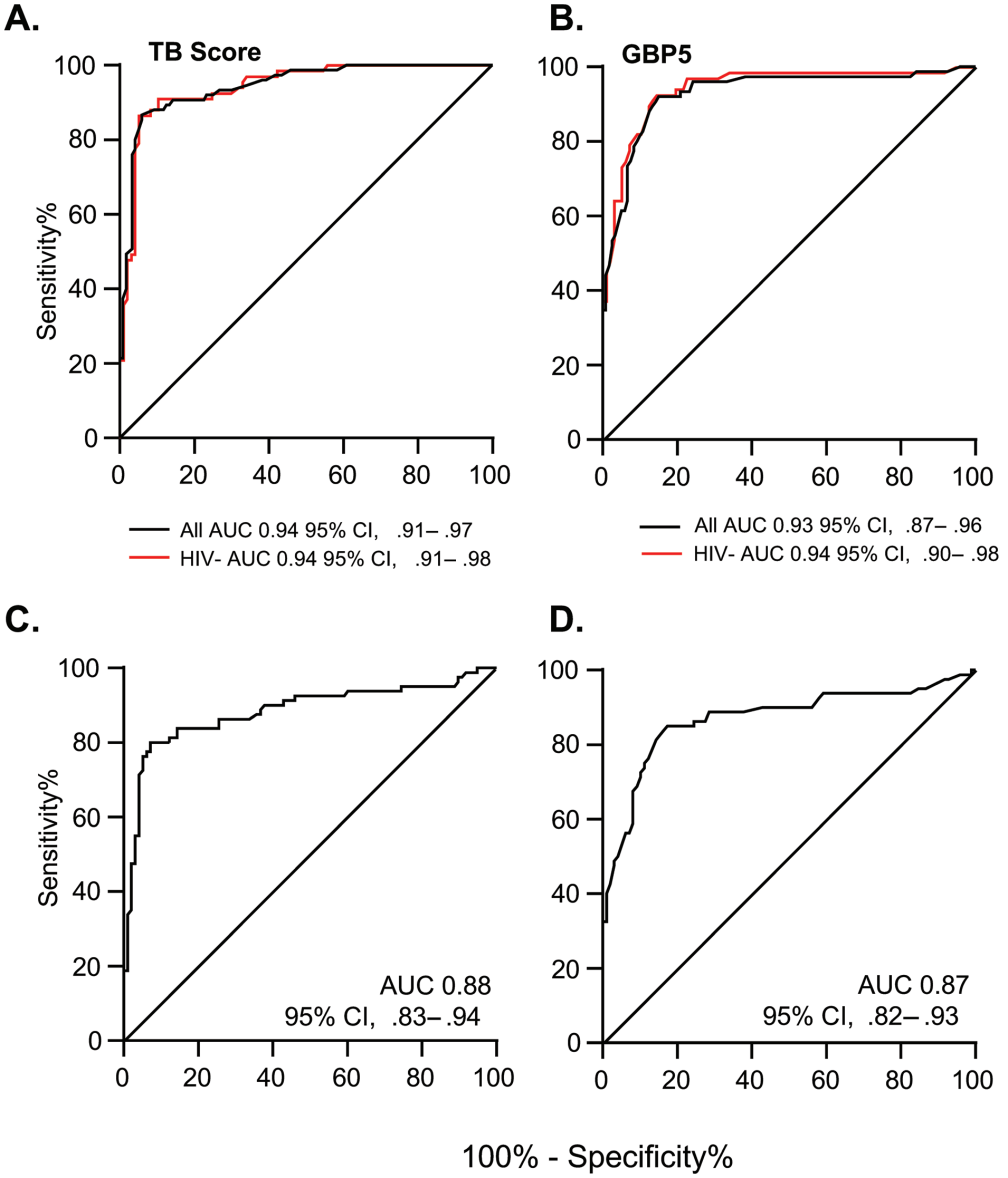
Xpert MTB-HR prototype analysis of all participants combined against Xpert Ultra. *A*, ROC curve for TB score all participants (*black line*) and participants who do not have HIV only (*green line*). *B*, ROC curve for GBP5 all participants (*black line*) and participants who do not have HIV only (*green line*). *C*, All participants evaluated against a composite microbiological score for TB score. *D*, All participants evaluated against a composite microbiological score for GBP5. Abbreviations: AUC, area under the curve; GBP5, guanylate binding protein 5; HIV, human immunodeficiency virus; MTB-HR, MTB-Host Response; ROC, receiver operator characteristic; TB, tuberculosis.

When Xpert-MTB-HR was evaluated against Xpert Ultra for the full cohort, the TB score discriminated between TB and ORD with an AUC of 0.94 (95% CI, .91–.97), sensitivity of 87% (95% CI, 77–93%), and specificity of 94% (88–97%) (Figure 2A, *black line*). Considering MTB-HR as a triage test, at a sensitivity of 90%, specificity was 86% (95% CI, 75–97%), which meets the minimal TPP criteria for a triage test. Further analysis revealed that GBP5 alone could discriminate between TB and ORD with an AUC of 0.93 (0.88–0.97) with a sensitivity of 90% and specificity of 86% (95% CI, 83–90%), thus also meeting the minimum TPP criteria for a triage test (Figure 2B, *black line*). Analysis of DUSP3 alone resulted in an AUC of 0.86 with a sensitivity of 89% (95% CI, 80–95%) but reduced specificity of 61% (95% CI, 52–69%) (data not shown). Thus, only results for TB score and GBP5 are shown for the remaining analyses.

When GeneXpert Ultra trace results were excluded (n = 68 TB, 120 ORD), we found a slightly higher AUC for the TB score of 0.95 (95% CI, .92–.98) and for GBP5 of 0.94 (95% CI, .90–.98) (data not shown). When participants with HIV were excluded (n = 67 TB, 97 ORD), there was no change in the AUC for the TB score (Figure 2A, *green line*) or GBP5 alone (Figure 2B, *green line*), with a sensitivity of 91% for both and specificity of 83% (95% CI, 70–96%) for the TB score and 86% (95% CI, 80–92%) for GBP5.

When the Xpert-MTB-HR was evaluated against a composite microbiological score (n = 80 TB, 98 ORD), the TB score was able to discriminate between TB and ORD with an AUC of 0.88 (95% CI, .83–.94), a sensitivity of 80% (95% CI, 76–85%) and a specificity of 94% (95% CI, 91–96%). Setting sensitivity to 90% for a triage test, specificity reduced to 58% (95% CI, 48–68%). GBP5 alone was able to discriminate between TB and ORD with an AUC of 0.87 (95% CI, .81–.93), a sensitivity of 86% (95% CI, 77–92%), and specificity of 76% (95% CI, 66–83%) (Figures 2C and 2D).

### Analysis of Individual Sites

**Figure 3.**
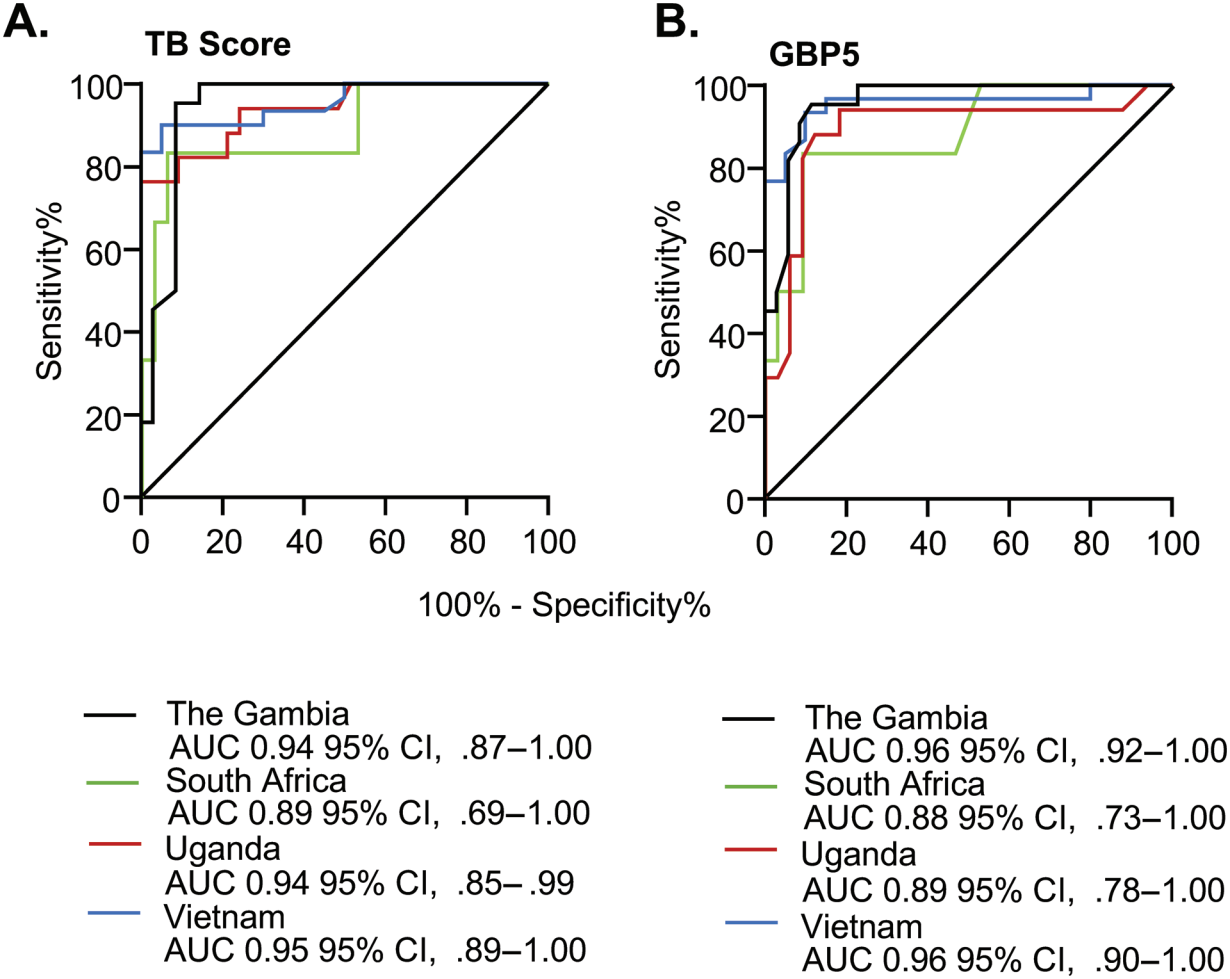
Evaluation at individual sites against Xpert Ultra. ROC curves are shown for Gambia (*black line*), South Africa (*green line*), Uganda (*red line*), and Vietnam (*blue line*). Abbreviations: AUC, area under the curve; CI, confidence interval; ROC, receiver operator characteristic.

When the TB score was evaluated against GeneXpert Ultra for Gambian samples (n = 22 TB, 35 ORD), we found an AUC of 0.94 (95% CI, .87–1.00) (Figure 3A, *black line*). Considering Xpert-MTB-HR as a triage test, at a sensitivity of 94%, specificity was 91% (95% CI, 86–96%). When GBP5 was analyzed alone, we found an AUC of 0.96 (95% CI, .91–1.00) (Figure 3B, *black line*) with a sensitivity of 91% (95% CI, 72–98%) and specificity of 91% (95% CI, 78–97%). When the TB score was evaluated against a composite microbiological score (n = 25 TB, 31 ORD), we found an AUC of 0.83 (95% CI, .70–.96) with a sensitivity of 88% (95% CI, 70–96%) and a specificity of 84% (95% CI, 67–93%).

When the TB score was evaluated against GeneXpert Ultra for South African samples (n = 6 TB, 32 ORD), we found an AUC of 0.89 (95% CI, .69-1.00) (Figure 3A, *green line*). Considering Xpert-MTB-HR as a triage test, at a sensitivity of 95%, specificity was 69% (95% CI, 31–100%). When GBP5 was analyzed alone, we found an AUC of 0.88 (95% CI, .73–1.00) (Figure 3B, *green line*) with a sensitivity of 83% (95% CI, 44–99%) and specificity of 91% (95% CI, 76–97%). When the TB score was evaluated against a composite microbiological score (n = 6 TB, 32 ORD), we found an AUC of 0.89 (95% CI, .73–1.00) with a sensitivity of 83% (95% CI, 44–99%) and specificity of 94% (95% CI, 80–99%).

When the TB score was evaluated against GeneXpert Ultra for Ugandan samples (n = 17 TB, 33 ORD), we found an AUC of 0.94 (95% CI, .85–.99) (Figure 3A, *red line*). Considering Xpert-MTB-HR as a triage test, at a sensitivity of 93%, specificity was 78% (95% CI, 67–89%). When GBP5 was analyzed alone, we found an AUC of 0.89 (95% CI, .78–1.00) (Figure 3B, *red line*) with a sensitivity of 94% (95% CI, 73–100%) and specificity of 82% (95% CI, 66–91%). When the TB score was evaluated against a composite microbiological score (n = 18 TB, 32 ORD), we found an AUC of 0.89 (95% CI, .79–1.00) with a sensitivity of 89% (95% CI, 67–98%) and specificity of 75% (95% CI, 58–87%).

When the TB score was evaluated against GeneXpert Ultra for Vietnamese samples (n = 30 TB, 20 ORD), we found an AUC of 0.95 (95% CI, .89–1.00) (Figure 3A, *blue line*). Considering Xpert-MTB-HR as a triage test, with a sensitivity of 92%, specificity was 83% (95% CI, 57–100%). When GBP5 was analyzed alone, we found an AUC of 0.96 (95% CI,.90–1.00) (Figure 3B, *blue line*) with a sensitivity of 93% (95% CI, 79–99%) and specificity of 90% (95% CI, 70–98%). When the TB score was evaluated against a composite microbiological score (n = 31 TB, 17 ORD), we found an AUC of 0.90 (95% CI, .82–.99) with a sensitivity of 84% (95% CI, 67–92) and specificity of 88% (95% CI, 66–98%).

## DISCUSSION

This is the first study to our knowledge to perform prospective point-of-care analysis of the Xpert-MTB-HR-Prototype using fingerstick blood from patients presenting with symptoms suggestive of TB but prior to microbiological confirmation. Our interim results show that the Xpert-MTB-HR prototype test meets the minimal criteria set out in the TPP for a triage test when evaluated against GeneXpert Ultra. This was a multi-site study within Africa and South-East Asia, and despite different host genetics, circulating Mtb strains (*M. Beijin*g in Vietnam, *M. tuberculosis sensu stricto* and *M. africanum* in sub-Saharan Africa) and comorbidities, all sites reached the TPP for a triage test. We also found that a single gene, GBP5 had the same sensitivity and specificity for TB among total respiratory infections compared to TB score, allowing for potential further simplification of the analytical process.

Surprisingly, there were very few differences in the performance of the cartridges seen between the sites. South Africa had the lowest AUC but this is likely due to the lower number of participants (only 6 in the TB arm) and is expected to change following full recruitment of 1200 participants. The Vietnam site performed similarly to sites in Africa suggesting the cartridge is applicable for global use, regardless of ethnicity, and underlying epidemiology of the region. This is of importance because the RNA signatures that provided the basis for the test, were mainly derived from sub-Saharan African cohorts. Both Gambia and Vietnam reached ideal specifications for a triage test when evaluated against GeneXpert Ultra. The accuracy of the prototype was reduced when evaluated against sputum culture as seen previously [10], with specificity lower than minimal criteria for a triage test when sensitivity was set at 90%. This is likely due to the smaller number of participants with available culture results. Our interim results suggest the Xpert-MTB-HR prototype could be used as a screening tool for individuals who cannot produce sputum or who have paucibacillary disease (ie, extrapulmonary TB, children) and would drastically reduce the biohazards associated with sputum production and culturing in the first instance. Although the performance of the cartridges may change following evaluation of our full cohort (including children), we felt it was important to publish these interim results in order to publicize one of the most promising triage tests for TB and the first one to reach the WHO TPP in the world. Given the very narrow 95% CI for all sites, and the similarity of performance between diverse study populations (including PLHIV), it is likely our performance will be retained due to our robust study design. However, we did not include cutoff values as these are likely to change following full evaluation of our cohort and evaluation by Cepheid of all data generated by the multiple consortia analyzing the prototype prior to commercialization.

The major limitation of this study is the relatively small sample size; however, our strengths were the use of a multisite study, inclusion of participants with HIV and those without, prospective analysis, and an appropriate control group of patients presenting with respiratory symptoms suggestive of TB but who were determined to have another respiratory infection. This allowed us to control for nonspecific effects of inflammation and is particularly important in analysis of the 3-gene signature, which will be affected by host inflammation. Indeed, the elevation of the GBP5 and DUSP3 pro-inflammatory genes with no difference in the anti-inflammatory KLF2 gene in the TB group shows that despite all patients presenting with a respiratory infection, the pro-inflammatory response associated with these biomarkers is greater in TB than other infections. We are currently determining which pathogens are present in nasopharyngeal swabs from the non-TB group as this may help to determine if we are able to achieve high discrimination between infections versus non-infectious disease diagnoses (ie, COPD).

The use of fingerstick blood for the Xpert-MTB-HR prototype was a major strength as previous studies have used stored Paxgene RNA samples. We transferred the blood to an EDTA minivette which precluded any issues with clotting prior to addition to the cartridge. It is likely that prior COVID infection may increase risk of progression to active TB disease as has been seen for prior influenza A virus infection [14] and will also be evaluated as part of our study. Importantly, HIV coinfection did not affect the accuracy of the test despite CD4 counts <500 in both groups. However, there was a borderline significant difference in TB score with lower median score in the individuals living with HIV and those who werenʼt within the ORD group.

In summary, our preliminary data show great promise for the use of the Xpert-MTB-HR-Prototype fingerstick blood test for screening of TB patients at the point-of-care. The ability to accurately discriminate based on a single gene (GBP5) suggests the potential for further simplification of the test. Although these findings are based on small participant numbers and small subgroups, they justify further evaluation.

## Notes

***Author contributions.*** J. S. S, and G. W. conceived and designed the study. J. S. S., G. W., T. N. T. T., H. M. K., H. M. D., J. W., T. J. S., A. Ge., and P. C. obtained funding. A. Gi., O. O., A. N., M. N., T. R., J. S., and S. T. M. developed protocols, recruited participants, and collected data. G. V. D. S. and K. S. designed the database. B. S. and J. W. provided project support. J. S. S. wrote the original draft, and J. S. S., G. W., T. N. T. T., H. M. K., H. M. D., T. J. S., A. Ge., J. W., S. T. M., J. S., and T. R. reviewed and edited manuscript drafts. J. S. S. and G. V. D. S. verified the underlying data.

***Acknowledgments.*** The authors first acknowledge all of the participants and their families. They also acknowledge Prof, Helena Kuivaniemi for project management and Prof, Purvesh Khatri for critical review of the manuscript. They also acknowledge the contribution of all TrENDx-TB consortium members: **MRCG:** Jayne Sutherland, Olumuyiwa Owolabi, Amie Secka, Georgetta Daffeh, Awa Gindeh, Joseph Mendy, Binta Sarr, Abi-Janet Riley, Alhaji Jobe, Monica Davies, Kairaba Kanyi, Momodou Jallow, Salieu Barry, Ousainou Cham, and Esin Nkereuwem; **SUN:** Gerhard Walzl, Stephanus Malherbe, Bronwyn Smith, Gian van der Spuy, Kim Stanley, Jane Shaw, Alicia Chetram, Tracy Richardson, Marika Finn, Andriette Hiemstra, Novel Chegou, Helena Kuivaniemi, Gerard Tromp, Susanne Tonsing, Elizma Smit, and Balie Carstens; **MAK:** Harriet Mayanja-Kizza, Mary Nsereko, AnnRitah Namuganga, Sophie Nalukwago, Joseph Akol, Dorcas Lamunu, and Michael Ordie; **OUCRU:** Guy Thwaites, Thuong Nguyen, Van Le, Son Vo Thanh, Hau Nguyen Thi, Ha Vu Thi Ngoc, and Ngoc Le Hong **CSU:** John Belisle and Karen Dobos **LSHTM:** Hazel Dockrell **SATVI:** Thomas Scriba, Mark Hatherill, Kate Hadley, Justin Shenje, Stanley Kimbung, Humphrey Mulenga, Rachel Oelofse, Nicole Bilek; Elma van Rooyen; and Simba Mabwe **LUMC:** Paul Corstjens, Annemieke Geluk, Elisa Tjon Kon Fat, Louise Pierneef, and Anouk van Hooij **Catalysis:** Jill Winter **FIND:** Morten Ruhwald, Emmanuel Moreau, and Adam Penn-Nicholson; LINQ: Claudia Schacht, Julia Büech, and Malte Streitz.

***Financial support.*** This study was jointly funded by European and Developing Countries Clinical Trial Partnership (EDCTP) (RIA2018D-2499; PI G. Walzl), National Institutes of Health (NIH) (grant number U01AI152075; PIs G. Walzl, J. Sutherland, J. Belisle) and the Bill & Melinda Gates Foundation (grant number INV-023825). Under the grant conditions of the Foundation, a Creative Commons Attribution 4.0 Generic License has already been assigned to the Author Accepted Manuscript version that might arise from this submission.

***Potential conflicts of interest. *** The authors: No reported conflicts of interest. All authors have submitted the ICMJE Form for Disclosure of Potential Conflicts of Interest. Conflicts that the editors consider relevant to the content of the manuscript have been disclosed.
